# Validation of L2 grit among Chinese EFL high school students and its enduring effect on achievements: A bifactor model approach

**DOI:** 10.3389/fpsyg.2022.971495

**Published:** 2022-09-29

**Authors:** Eerdemutu Liu, Junju Wang, Sachurina Bai

**Affiliations:** ^1^School of Foreign Language and Literature, Shandong University, Jinan, China; ^2^English Department, Hohhot No. 2 Middle School, Hohhot, China

**Keywords:** L2 grit, language achievement, bifactor confirmatory factor analysis (bifactor CFA), exploratory structural equation modeling (ESEM), bifactor exploratory structural equation modeling (bifactor ESEM), measurement invariance

## Abstract

The current study seeks to validate L2 grit measure among 637 Chinese senior middle school students using a bifactor modeling approach. To do so, we first assessed and compared four alternative measurement models including CFA, bifactor CFA, ESEM, and bifactor ESEM models. Among these models, CFA exhibited the poorest fit to the data collected from the sample. ESEM showed partial fit to the data with a slightly lower factor correlation between two components of L2 grit (i.e., perseverance of effort and consistency of interest) than CFA. Two bifactor models (bifactor CFA and bifactor ESEM) demonstrated excellent fits to the data. The more parsimonious bifactor CFA model was selected as the optimal one. Based on the bifactor CFA model, we confirmed measurement invariance across gender and predictive validity of L2 grit on subsequent language achievements. Based on these findings, methodological and pedagogical implications were discussed.

## Introduction

Language learning needs long-term dedication to succeed. Hence, a large amount of language learners falls short of success, leading L2 researchers to investigate the factors that positively influence language learning outcomes. With the introduction of positive psychology in the SLA field, a host of positive attributes have emerged as crucial determinants in language learning outcomes (Macintyre et al., 2019; Wang et al., 2021). Grit was postulated as a non-cognitive, relatively stable individual difference factor and was conceptualized as passion and perseverance for long-term goals (Duckworth et al., 2007). It was even claimed to have a more powerful predictive effect than IQ and aptitude on achievement (Duckworth, 2013). Given this, it is not surprising that there is an exponential growth in grit research in language learning domain. As noted by Teimouri et al. (2021), it may be the individual difference factor that received the most widely research attention in language learning field in recent years. Adopting the general grit scale, recent empirical research revealed that grit can be a crucial predictor of language learning outcomes such as motivation, emotions, willingness to communicate, and language achievement (e.g., Khajavy et al., 2021; Lan et al., 2021; Li and Dewaele, 2021; Liu and Wang, 2021). However, using the domain-general scale in a domain-specific field can bias the construct validity and predictive validity (Teimouri et al., 2021). One, for example, can be a gritty person regarding overall learning except for foreign language learning simply due to its lengthy process of memorizing a large amount of vocabulary and grammar. That is, similar to anxiety, grit has the feature of domain-specificity (Schmidt et al., 2017; Cormier et al., 2019; Teimouri et al., 2021).

Accordingly, Teimouri et al. (2020) adapted the grit scale to the language learning field. But they used only principal component analysis, which may be not enough to confirm the factor structure of L2 and risk losing other important information for further testing of the model (Marsh et al., 2014; Alamer and Marsh, 2022). The traditional CFA model to test the factor structure has its own flaws; it is viewed as an over-restrictive model by setting no cross-loading and often followed by correlating item residuals or deleting items, a problematic data-driven method that can distort the original meaning of the well-established construct (Marsh et al., 2014; Morin et al., 2016). This was evidenced by the subsequent research that tested the factor structure of L2 grit using CFA, resulting in poor model fits (e.g., Sudina et al., 2020; Elahi Shirvan et al., 2021; Sudina and Plonsky, 2021). In contrast to CFA, ESEM has the advantages of allowing for cross-loadings while producing model fit indices information (Morin et al., 2020). Bifactor model, on the other hand, can test the global and specific factors simultaneously, and failing to account for the global factors when it exists may inflate the factor loadings and factor correlations (Morin et al., 2016, 2020).

Thus, in the current study, we seek to test the factor structure of L2 grit in a more comprehensive and systematic way using more recent developments in statistical analysis. More specifically, we seek to test and compare four alternative models – CFA, bifactor CFA, ESEM (exploratory structural equation modeling), and bifactor ESEM to identify the optimal model to represent the factor structure of L2 grit. In addition, measurement invariance of the selected L2 grit model across gender was tested given that gender has long been incorporated as an important factor influencing language learning (e.g., Green and Oxford, 1995; Dewaele et al., 2016), and measurement invariance is an important prerequisite to the comparison across groups (Chen et al., 2005; Schmitt and Kuljanin, 2008; Sass and Schmitt, 2013). Finally, we tested the predictive validity by relating L2 grit with language achievement. We are particularly interested in the enduring effect of grit on subsequent consecutive language achievements. As grit features the sustained effort and passion for long-term goals, it is reasonable to assume that the persistent effort and interest can have a lasting effect on language learning achievements. Therefore, the aims of the current study are three-fold: (1) to test the factor structure of L2 grit using four alternative models (i.e., CFA, bifactor CFA, ESEM, and bifactor ESEM); (2) to test the measurement invariance of L2 grit; (3) to examine the predictive validity of L2 grit in subsequent three language achievements.

## Literature review

### Domain-general grit

Grit, a higher-order construct with two facets – perseverance of effort and consistency of interest – denotes the commitment to long-term goals with sustained effort and interest (Duckworth et al., 2007). According to Duckworth (2016), it is an important personality trait to predict lifetime success especially over the long run. But it is distinct from conceptually related constructs such as Big Five conscientiousness, as grit denotes not only working hard on the current work, but also working arduously towards the long-term goal for an extended period of time (Duckworth et al., 2007; Eskreis-Winkler et al., 2014). In addition, it has been empirically demonstrated that grit can predict success over and above Big Five conscientiousness (Duckworth et al., 2007; Duckworth and Quinn, 2009; Eskreis-Winkler et al., 2014). In their pioneering studies, Duckworth and her colleagues found that grit can predict educational attainment among adults, grade point average among undergraduates, retention of cadets after training, ranking in National Spelling Bee test, less career change, and less time spent on television (Duckworth et al., 2007; Duckworth and Quinn, 2009). Grit has also been found to be relevant to job performance (Ion et al., 2017; Mueller et al., 2017) and teacher effectiveness (Robertson-Kraft and Duckworth, 2014). In their meta-analysis with 83 studies involving 66,518 participants, Hou et al. (2021) has revealed the positive relation of grit with subjective well-being.

More relevant to the current study is the learning achievement in school setting. Although it seems intuitively that grit is related to academic success, research findings were mixed. While some found that grit was strongly related to learning outcomes (e.g., Duckworth et al., 2007; Duckworth and Quinn, 2009; Strayhorn, 2014), others revealed only weak relation (e.g., Li et al., 2018; Steinmayr et al., 2018). Some others suggested that grit could predict achievement in the first step, but not when controlling for the effect of previous achievement (e.g., Chang, 2014). In their meta-analysis study based on 88 studies, Credé et al. (2017) revealed that grit was positively and weakly related to academic performance. They proposed a set of possible moderating factors that may attenuate the grit-achievement relation such as task difficulty and grit level. For example, too easy task may retrain one to show grit, and when one is over-gritty, they may not seek help from others, thus weakening the grit-achievement relation. In addition, they also questioned the construct validity of grit as a higher-order construct, as the two components of perseverance and passion were not strongly correlated, and the facet of perseverance demonstrated a higher predictive power than passion. Similarly, Lam and Zhou’s (2019, 2022) meta-analyses reported that overall grit as well as its two factors of perseverance and passion were both significantly and positively related with academic achievement. In addition, they found that the perseverance of effort exhibited the strongest relation with academic achievement followed by overall grit and consistency of interest. Although it seems that the role of domain-general grit in general academic achievement is well-established, we cannot simply assume that these findings can be applied to a domain-specific language learning field (Teimouri et al., 2021), which warrants research into how grit affect language learning.

### Domain-specific grit

In the SLA field, grit has captured the attention of researchers only recently. Language learning is a lengthy process that needs persistence and perseverance on the part of language learners (MacIntyre and Khajavy, 2021). Thus, it is reasonable to assume that grit – the passion and perseverance for long-term goals–can be an important contributor to motivational behavior and language achievement (Teimouri et al., 2021). However, much recent research that examined grit using a domain-general scale has yielded inconsistent findings (Yamashita, 2018; Robins, 2019, Unpublished doctoral dissertation^1^; Khajavy, 2021; Liu and Wang, 2021; Thorsen et al., 2021; Khajavy and Aghaee, 2022). According to Teimouri and his associates, this may be due to the use of domain-general measure in the domain-specific context of language learning (Teimouri et al., 2020, 2021). For example, individuals can be gritty with regard to all subjects except for language learning simply because language learning needs memorizing a large amount of vocabulary and grammar. Therefore, Teimouri et al. (2020) developed a domain-specific L2 grit scale, which has become the most widely used L2 grit scale (e.g., Sudina et al., 2020; Wei et al., 2020; Elahi Shirvan et al., 2021; Sudina and Plonsky, 2021). In addition, they convincingly argued that using both domain-specific measures of grit and achievement enhanced the construct and predictive validity of L2 grit (Teimouri et al., 2021).

Although recognizing their important contribution to the grit literature in L2 field, we identified one important limitation – they used only principal component analysis (PCA) to assess the factor structure of L2 grit. PCA may be insufficient for supporting construct validity in that it cannot provide information on the latent variables with corrected measurement errors and cannot be used for further analysis such as the structural relations between latent constructs, invariance across multigroup, and comparison of competing models (Marsh et al., 2014; Alamer and Marsh, 2022). Moreover, it is primarily used for data reduction but not for interpretation (Hair, 2019). Accordingly, subsequent research that assessed the factor structure using CFA often resulted in poor model fit (e.g., Sudina et al., 2020; Elahi Shirvan et al., 2021; Sudina and Plonsky, 2021). Thus, we seek to evaluate the factor structure of L2 grit in a more comprehensive way by using more advanced measurement models (bifactor CFA, ESEM, and bifactor ESEM), which will be described in the next section.

As part of the validation of L2 grit, we tested the measurement invariance across gender. Gender has always been regarded as an important factor in language learning. Accordingly, it is a common practice to compare constructs across genders, such as language learning strategies (Green and Oxford, 1995), anxiety and enjoyment (Dewaele and MacIntyre, 2014), and learning style (Bessonova and Misbakhova, 2019). Similarly, L2 grit has also been compared across gender (e.g., Wei et al., 2020). However, to date, no study has reported the measurement invariance of the L2 grit across gender, one important prerequisite to the comparison across the group. That is, only when the measure has the same meaning across gender, can we use the measure to compare across gender (Chen et al., 2005; Schmitt and Kuljanin, 2008; Sass and Schmitt, 2013).

With regard to the effect of grit on achievement in language learning field (i.e., predictive validity), a few recent studies using domain-specific grit has reported more consistent, robust findings than those using domain-general grit (Teimouri et al., 2021). For example, both Teimouri et al. (2021) and Sudina and Plonsky (2021) found domain-specific grit to be a positive predictor of achievement and reported domain-specific grit to be positive predictor of L2 achievement over and above domain-general grit, confirming the superiority of the use of domain-specific construct. However, less consistent is the relative predictive power of perseverance and passion on L2 achievement. Whereas Teimouri et al. (2021) found perseverance as the stronger predictor of L2 achievement, Sudina and Plonsky (2021) identified consistency of interest as the more robust predictor of L2 achievement. Despite these recent studies on the role of grit in L2 achievement, little research has examined the lasting effect of L2 grit on language achievement. As grit was conceptualized as passion and perseverance for long-term goals (Duckworth et al., 2007; Duckworth and Quinn, 2009; Teimouri et al., 2020), we hypothesize that these passion and perseverance for long-term goals not only influence short-term language achievement but also have an enduring effect on language achievement. One exception is the study by Alamer (2022b) that used data from Saudi undergraduate English majors and adapted the consistency of interest to single language interest to exclusively examine how it interacts with motivation orientations (autonomous vs. controlled motivation) to influence language achievements. He found that initial single language interest at time 1 predicted later single language interest at time 2 which in turn predicted language achievement at time 3 after controlling for the effect of achievement at time 1. Additionally, they revealed that the two distinct types of motivational orientation differentially moderated the effect of single language interest on language achievement. These finings also supported our hypothesis of long-term effect of grit on subsequent language achievements. The present study is different from the study by Alamer (2022b) in that we included both consistency of interest and perseverance of effort as well as the global grit in our model and assessed their enduring effect on three subsequent language achievements among Chinese high school students.

### The alternative models of ESEM, bifactor CFA, and bifactor ESEM

The traditional approach to examining the construct validity is exploratory factor analysis (EFA) or principal component analysis (PCA) followed by confirmatory factor analysis (CFA) (Kelloway, 2014). EFA and PCA are typically used to extract a smaller number of latent factors to represent a larger number of items especially when no *a priori* assumption was made concerning the factor structure of the construct of interest (Plonsky, 2015). However, they are limited when used to assess psychometric validity, as it does not yield other important values such as goodness-of-fit indices as in CFA. CFA has the advantage of producing goodness of fit indices that allows for comparison of competing models, multi-group invariance analysis, and autoregressive path model, to name a few (Marsh et al., 2014). But in CFA, the cross-loadings are constrained to be zero standing in sharp contrast with EFA that allows for freely-estimated cross-loadings (Asparouhov and Muthén, 2009; Marsh et al., 2014; Morin et al., 2016). This distinction often results in poor model fit in CFA and subsequently be modified by correlating multiple measurement errors or deleting items. However, the data-driven model modification has been criticized for having no theoretical ground (Asparouhov and Muthén, 2009; Marsh et al., 2014). For example, correlating multiple measurement errors would lead one to speculate that one or more additional factors may not be adequately captured by the model. In addition, the constraint of zero cross-loadings is over-restrictive and unrealistic and causes over-estimation of factor correlations and a distorted structural model that tests the relationships between constructs (Asparouhov and Muthén, 2009; Marsh et al., 2009, 2014; Schmitt and Sass, 2011).

Given the problem noted above, more viable and flexible approaches were proposed including ESEM, bifactor CFA, and bifactor ESEM (Howard et al., 2016; Morin et al., 2016). Exploratory structural equation modeling (ESEM) integrates the advantages of EFA and CFA by allowing non-zero cross-loadings between items and non-target factors and providing model fit indices (Morin et al., 2020). Distinct from EFA and CFA which can be viewed as both confirmatory or exploratory approaches, ESEM is seen as primarily a confirmatory one as indicated in the use of target rotation (Marsh et al., 2014). ESEM has been reported to provide a better fit to the data and can be directly compared with CFA results (e.g., Alamer, 2022a).

Both CFA and ESEM can test the bifactor model. Past research that examined the dimensionality of constructs was faced with a choice between overall factor (e.g., grit) or specific factors (e.g., perseverance of effort and consistency of interest). With the development of psychometric assessment, the bifactor model has been introduced to assess both overall and specific factors simultaneously (Morin et al., 2016). In the bifactor model, the overall factor and specific factors are set to be orthogonal (unrelated), and the specific factors account for the remaining shared variance not explained by overall factor (Morin et al., 2020). The bifactor model may provide unique information, such that specific factors may provide additional or differential information than the global factor (Alamer, 2022a), and ignoring the global factor when it exists may result in overestimation of cross-loadings in ESEM or CFA and factor correlations (Morin et al., 2016, 2020). The bifactor model can be used with a higher-order construct and ESEM can be employed with a construct with related factors (Morin et al., 2020). L2 grit, like its domain-general grit, meets these criteria, as it was conceptualized as a two-layer construct – global grit in the second layer with two related factors – perseverance of effort and consistency of interest in the first layer (Teimouri et al., 2020). Therefore, we selected CFA, bifactor CFA, ESEM, and bi-factor ESEM as alternative models to examine the construct validity of L2 grit. Although second-order and bifactor models are both higher-order models, the second-order model was excluded in our analysis, as it has been criticized for being linked to indicators only indirectly *via* first-order factors (Howard et al., 2016; Alamer and Marsh, 2022) and a second-order higher-order CFA model with two correlated sub-components (i.e., perseverance of effort and consistency of interest) will be statistically the same with the first-order CFA (Alamer, 2021). This is in contrast to the bifactor model that links both general and specific factors directly to the items. In addition, bifactor model is more appropriate when researchers are interested in the contributions of specific factors over and above the general factor, as this model forces the factor correlations to be orthogonal (i.e., uncorrelated) (Chen et al., 2006).

Only one research has adopted a bifactor modeling approach to examine the construct validity of L2 grit. Being aware of the methodological problems of Teimouri et al. (2021) L2 grit scale that used only PCA to confirm its validity, Alamer (2021) employed a more rigorous method of EFA followed by CFA and bifactor CFA to test the validity of the L2 grit scale. He found that bifactor CFA but not CFA fitted the data. In addition, they revealed that grit was related to the ideal self, motivational intensity, and controlled motivation. Based on the bifactor model, L2 grit was linked with vocabulary knowledge, concluding that the two factors of perseverance of effort and consistency of interest exhibited predictive power over and above grit.

The current study is unique in that (1) the validity of L2 grit was assessed among Chinese senior middle school students by comparing more alternative models (CFA, bifactor CFA, ESEM, and bifactor ESEM); (2) measurement invariance of the model was tested across gender; and (3) predictive validity was evaluated by examining the enduring effect of L2 grit on language achievement.

## Methodology

### Research sample

The sample consisted of 637 first-year students from a key senior middle school from northern China. Among them, 305 were girls and 332 boys, aged between 16–18. They followed the same curriculum and used the same textbook. On average, they had six 40-min English classes each week. During each term, they attended at least a monthly test, a mid-term test, and a final test, following the norms of the National College Entrance Examination. None of them had the experience of traveling or studying in a native-English country.

### Research instruments

#### L2 grit scale

Students’ level of perseverance of effort and consistency of interest in language learning was assessed using the Chinese version of L2 grit scale (Teimouri et al., 2020), which was adapted from the original domain-general grit scale (Duckworth et al., 2007). Similar to the domain-general grit scale, L2 grit consists of two dimensions, consistency of effort (4 items) and perseverance of effort (5 items). All the items in the consistency of effort were negatively worded and thus reverse-coded. Sample items include “I am a diligent English language learner,” and, “I think I have lost interest in learning English.” Students responded to the items on a 5-point Likert scale. The Chinese version of the L2 grit scale was obtained by using translation and back-translation method by two independent Chinese-English bilingual teachers (see Appendix 1 in Supplementary material). The internal consistencies of overall grit, perseverance of effort, and consistency of interest as indicated by Cronbach’s alphas were 0.786, 0.724, 0.727, respectively.

#### Language achievement test

Students’ three English test scores were obtained to indicate their English levels at the respective time point. The three English tests included three mid-term tests with 6-month interval. Students attended the initial mid-term test 2 weeks after they finished the questionnaire. All the English tests followed the patterns and norms of college entrance examination, a high-stake test in China. The test consists of six parts, namely listening (30 points), reading (40 points), cloze (30 points), fill-in-blanks (15 points), error correction (10 points), and essay writing (25 points). As in the English test in college entrance examination in some less-developed provinces in China (typically National Volume B) including the province where the participants’ schools were located, the listening score was not included in the total score with the initial maximum score of 120, which was then multiplied by 1.25. Thus, the final total score was 150. All the tests were administered in a paper-and-pen format within 90 min. The internal consistency of each of the three tests were 0.824, 0.847, 0.861, respectively.

### Data collection procedure and analysis

First, we obtained the approval from the headteacher, class teachers, and English teachers to administer the questionnaire and collect students’ test scores. Then, we obtained informed consent from students’ parents or legal guardians *via* the WeChat group, a social media messaging app popular in China. Before administering the questionnaire survey, we informed students of the aims and confidentiality of the current study. After the questionnaire, we collected students’ three consecutive test scores, including one monthly test and two mid-term test scores.

All the data analysis was performed using Mplus (8.3) except for the correlational analysis which was carried out by SPSS (26) (see Mplus codes in Appendix 2 in Supplementary material). MLR estimator was used to address the potential non-normality problem. McDonald’s composite reliability (ω) (McDonald, 1970) was used to assess and compare the internal consistencies of four measurement models of L2 grit. It is different from traditional Cronbach alpha reliability in that it takes into account the factor loadings and error measurements, thereby capturing the nuanced differences between four measurement models (Peterson and Kim, 2013).

The internal structure of the L2 grit was assessed by using and comparing four model fit statistics of CFA, bifactor CFA, ESEM, and bifactor ESEM. Multiple indices were used to assess and compare the goodness of fit of the models, including CFI (confirmatory fit index), TLI (Tucker-Lewis index), RMSEA (root mean square error of approximation), and SRMR (standardized root mean square residual). Chi-square statistics was not used as it tends to increase with sample size (Kline, 2015). CFI and TLI larger than 0.9 and 0.95, RMSEA and SRMR less than 0.08 and 0.06 were acceptable and excellent, respectively (Hu and Bentler, 1999; Marsh et al., 2004). To compare model fit indices, smaller than –0.01 change in CFI and 0.015 in RMSEA were used to indicate invariance between two models (Cheung and Rensvold, 2002; Chen, 2007).

**Figure 1:**
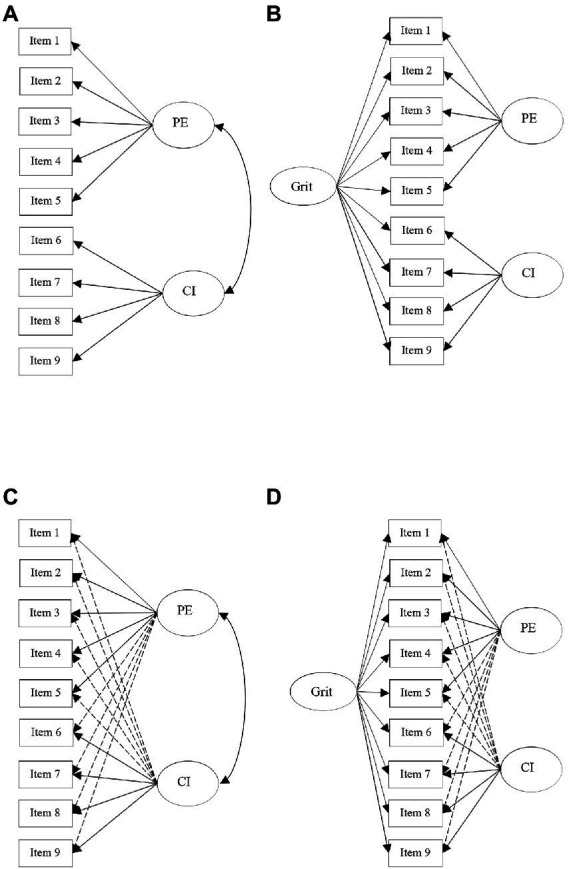
Four measurement models of L2 grit, **A** = CFA model; **B** = bifactor CFA model; **C** = ESEM model; **D** = bifactor ESEM model; PE = perseverance of effort; CI = Consistency of interest.

In the current study, four models were tested as follows (see Figure 1):

Standard CFA with items loaded on their target specific factors (Figure 1A).
Bifactor CFA with items loaded on specific target factors and one general factor with factors set to be orthogonal (uncorrelated) (Figure 1B).
Standard ESEM with items loaded on specific factors, allowing items to cross-load on their non-target factors. (Figure 1C).
Bifactor ESEM model with all items loaded on both specific factors and one general factor, allowing items to cross-load on their non-target factors and setting the factors to be orthogonal (uncorrelated; Figure 1D).

For measurement invariance, multi-group CFA was employed to test a set of nested models in an increasingly restrictive manner (Schmitt and Kuljanin, 2008; Byrne, 2013). First, we began by testing configural invariance to assess whether the same pattern of items and latent variables existed in two groups without any equality constraint. Second, weak or metric invariance was tested by constraining factor loadings to be equal across the groups. Third, scalar or strong invariance was performed by constraining item intercept to be equal across groups. Finally, strict invariance was tested to assess the equality of residual of indicators.

Then, based on the bifactor structural model, we tested the predictive validity of L2 grit by examining the relations of L2 grit and language achievements over three time period.

## Results

### The identification of optimal L2 grit measurement model

Tables 1, 2 report the model fits and factor loadings of the four models, respectively. Following the guidelines proposed by Morin and his colleagues (Morin et al., 2016, 2020; Alamer 2022a), we first compared CFA and ESEM. While CFA produced very poor fit to the data (CFI = 0854, TLI = 0.798, RMSEA = 0.114, SRMR = 0.085), ESEM demonstrated partial fit (CFI = 0.910, TLI = 0.830, RMSEA = 0.105, SRMR = 0.054). All the factor loadings in CFA loaded significantly (*p* < 0.05) and saliently (|*λ*| >0.3) on their target factors, except for the two items (PE3 and CI1), which were significant but not salient. All the factor loadings in ESEM were significant and salient except for three items, two (PE3 and CI1) loading significantly but not saliently on their target factors, and one (PE4) loading significantly and salient on its non-target factor. This indicated the violation of the assumption of the model structure. With regard to the reliability, as Table 2 shows, composite reliabilities of both models were within an acceptable range (*ω* = 0.731 to 0.764). Compared to CFA, ESEM produced less factor correlation (*r* = 0.479 for CFA, *r* = 0.460 for ESEM), meaning that ESEM allowing cross-loading reduces overestimation of factor correlation between two components of perseverance of effort and consistency of interest. Hence, the superiority of the ESEM model over the traditional CFA model regarding L2 grit was confirmed by the findings of the present study.

**Table 1:** Model fit indices for four measurement models of the L2 grit.

**Model**	***χ***^**2**^	***p***	***df***	**CFI**	**TLI**	**RMSEA**	**SRMR**
CFA	241.6	0.000	26	0.854	0.798	0.114	0.085
ESEM	151.2	0.000	19	0.910	0.830	0.105	0.054
Bifactor CFA	26.94	0.080	18	0.994	0.988	0.028	0.018
Bifactor ESEM	22.83	0.029	12	0.993	0.978	0.038	0.014

**Table 2:** Factor loadings of the four measurement models of L2 grit.

**Items**	**CFA**	**Bifactor CFA**		**ESEM**		**Bifactor ESEM**		
	β	S-β	G-β	β	β	S-β	S-β	G-β
PE1	0.82	0.429	0.694	**0.834**	−0.037	**0.455**	0.002	**0.692**
PE2	0.84	0.48	0.732	**0.887**	−0.053	**0.464**	−0.005	**0.728**
PE3	0.25	0.121	0.423	**0.147**	0.17	**0.135**	−0.04	**0.466**
PE4	0.43	0.142	0.735	**0.264**	0.303	**0.126**	0.062	**0.679**
PE5	0.55	0.116	0.544	**0.534**	0.012	**0.103**	−0.038	**0.556**
***ω***	**0.731**	**0.820**		**0.764**		**0.862**		
CI1	0.28	0.326	0.169	0.04	**0.262**	0.042	**0.331**	**0.164**
CI2	0.60	0.367	0.517	0.251	**0.477**	0.019	**0.368**	**0.522**
CI3	0.84	0.676	0.447	−0.005	**0.835**	−0.015	**0.686**	**0.442**
CI4	0.86	0.813	0.417	−0.072	**0.913**	−0.025	**0.808**	**0.411**
***ω***	**0.761**	**0.784**	**0.863**		**0.764**		**0.862**	**0.862**

S, specific factor; G, general factor; ω, composite reliability; PE, perseverance of effort; CI, consistency of effort; The bolded data are the standardized factor loadings on their target factors.

Then we compared bifactor CFA and bifactor ESEM model. Both bifactor CFA (CFI = 0.994, TLI = 0.988, RMSEA = 0.028, SRMR = 0.018) and bifactor ESEM demonstrated excellent fit to the data (CFI = 0.993, TLI = 0.978, RMSEA = 0.038, SRMR = 0.014) with the latter showing slightly less fit than the former. Both models produced acceptable reliabilities (bifactor CFA, *ω* = 0.820 for PE, 0.784 for CI, and 0.863 for overall grit; bifactor ESEM *ω* = 0.862 for PE, CI, and overall grit). On closer look at the factor loadings, it can be observed that those of bifactor CFA and bifactor ESEM were significant and salient on general factor and/or specific factors. Therefore, the bifactor CFA model was finally selected as the representation of the L2 grit construct and used for subsequent analyses. In the bifactor CFA model, the low factor loadings of PE3, PE4, and PE5 for the specific PE factor but their high factor loadings on global grit are acceptable as they indicate that these three items were primarily represented in the global factor of L2 grit rather than in the specific factor of perseverance of effort. The reverse is true for the item CI1 signifying that it is mostly represented by the specific item of consistency of effort rather than the global factor of L2 grit.

### Measurement invariance of L2 grit across gender

Table 3 shows the results of the measurement invariance test of L2 grit based on the bifactor CFA model. Configural invariance without any constraint showed excellent fit to the data (CFI = 0.987, TLI = 0.974, RMSEA = 0.041, SRMR = 0.026). Then weak invariance (equality on factor loadings), strong invariance (equality on factor loadings and intercepts), and strict invariance (equality on factor loadings, intercepts, and item uniqueness) were tested. All the invariance tests produced non-significant change (ΔCFI <− 0.01, ΔRMSEA < 0.015), supporting the invariance of L2 grit measures across gender.

**Table 3:** Measurement invariance test of L2 grit using bifactor CFA.

**Model**	***χ***^**2**^***(df)***	***p***	**CFI**	**TLI**	**RMSEA**	**SRMR**	**ΔCFI**	**ΔRMSEA**
Model 1	55.369 (36)	0.0205	0.987	0.974	0.041	0.026		
Model 2	70.396 (51)	0.0372	0.987	0.982	0.035	0.051	0.001	0.006
Model 3	86.055 (57)	0.0042	0.981	0.975	0.04	0.056	0.006	0.005
Model 4	94.582 (66)	0.0121	0.981	0.979	0.037	0.079	0.001	0.003

Model 1 = configural invariance; Model 2 = weak invariance; Model 3 = Strong invariance; Model 4 = strict invariance.

### Bifactor structural mode of L2 grit predicting language achievements over three time points

**Figure 2:**
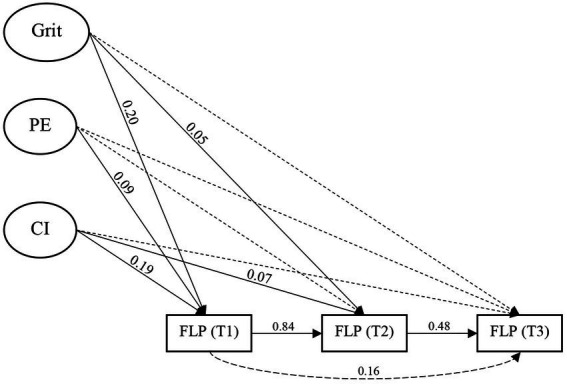
The enduring effect of L2 grit on subsequent three language achievements. The solid lines indicate significant relations and the dotted lines non-significant.

Before we test the structural model, we first examined the correlation among the key variables as the preliminary analysis. As Table 4 shows, all the key variables were significantly and positively related. Table 5 presents the results of the bifactor structural model of L2 grit predicting language achievement over three time period after controlling for the effect of gender and age. The model showed acceptable fit to the data (CFI = 0.973, TLI = 0.951, RMSEA = 0.034, SRMR = 0.023). As shown in Table 6 and visualized in Figure 2, among the three factors predicting the subsequent language achievement at T1, general grit exhibited the strongest predictive power (*β* = 0.20, *p* < 0.001), followed by consistency of interest facet (*β* = 0.19, *p* < 0.001), and then by perseverance of effort facet (*β* = 0.09, *p* < 0.05), supporting the predictive validity of grit in language achievement. In addition, the findings suggested that the two dimensions of perseverance of effort and consistency of interest exhibited predictive power over and above the general grit. This further supports the bifactor model adopted in the current study that incorporates both general L2 grit and its two dimensions. The language achievements at all three time-point were significantly related to each other except for the relation between T1 and T3, indicating that the achievement at T1 was primarily related to the achievement at T3 through achievement at T2. For the enduring effect of grit, both general grit and consistency of interest predicted students’ language achievement at T2 after controlling for the effect of T1. This suggests that the grit had the potential lasting effect on language achievement and the consistency of interest facet had the lasting effect on language achievement over and beyond the general grit.

**Table 4:** Correlation among key variables.

Variables	Grit T1	PE T1	CI T1	FLP T1	FLP T2	FLP T3
Grit T1	1					
PE T1	0.867^**^	1				
CI T1	0.835^**^	0.448^**^	1			
FLP T1	0.260^**^	0.181^**^	0.266^**^	1		
FLP T2	0.264^**^	0.184^**^	0.270^**^	0.854^**^	1	
FLP T3	0.278^**^	0.189^**^	0.289^**^	0.842^**^	0.859^**^	1

**p < 0.01; PE, perseverance of effort; CI, consistency of effort; FLP, Foreign Language achievement.

**Table 5:** Fit indices for the structural model based on bifactor CFA L2 grit predicting language achievement over time.

**Model**	***χ***^**2**^	***p***	***df***	**CFI**	**TLI**	**RMSEA**	**SRMR**
Structural Bifactor CFA	13757.13	0.00	90	0.973	0.951	0.034	0.023

**Table 6:** Structural model based on bifactor CFA predicting language achievements over time.

	**FLP T1**			**FLP T2**			**FLP T3**		
	*β*	SE	*p*	*β*	SE	*p*	*β*	SE	*p*
Grit	0.20	0.04	***	0.05	0.02	*	0.04	0.03	ns
PE	0.09	0.04	*	−0.01	0.03	ns	−0.05	0.03	ns
CI	0.19	0.05	***	0.065	0.02	**	0.03	0.02	ns

*p < 0.5; ***p < 0.001; PE, perseverance of effort; CI, consistency of effort, T, time; ns, not significant.

## Discussion

The present study seeks to identify the optimal model to represent the factor structure of L2 grit by comparing four measurement models – CFA, ESEM, bifactor CFA, and bifactor ESEM. Then, drawing on the optimal model of L2 grit, we conducted a measurement invariance test across gender and assessed its predictive validity in language achievement. In addition, we also tested the enduring effect of L2 grit on language achievements. Among the four models that we assessed, the CFA model exhibited the poorest fit and did not reach an acceptable level, which was in line with prior research that examined the construct validity of L2 grit using CFA (Sudina et al., 2020; Alamer, 2021; Elahi Shirvan et al., 2021; Sudina and Plonsky, 2021). The previous research typically employed the strategies of deleting items or correlating item residuals (e.g., Sudina et al., 2020; Elahi Shirvan et al., 2021; Sudina and Plonsky, 2021), which was considered a pure data-driven method and thus an untheoretical solution (Alamer and Marsh, 2022). Instead, the current study tested alternative three more advanced models (i.e., bifactor CFA, ESEM, and bifactor ESEM). Despite the partial fit, the ESEM model produced a better fit and lower factor correlation than CFA with both of them reaching acceptable composite reliabilities. This result indicates that the ESEM is a more advanced solution as it allows items to cross-load on their non-target factors producing higher fit and reducing inflated factor correlation between the perseverance of effort and consistency of interest, relative to CFA that sets over-restrictive zero cross-loadings, thereby yielding less fit and inflated higher correlation between the two factors. This advantage of ESEM over CFA has also been confirmed in other constructs such as passion and basic psychological needs in previous research within the SLA field (Alamer and Marsh, 2022; Alamer, 2022a).

Two bifactor models (bifactor CFA and bifactor ESEM) both reached an excellent fit with the data. This indicates that the bifactor model can better represent the L2 grit construct which assumes the existence of both overall L2 grit and specific factors of perseverance of effort and consistency of interest. In other words, these two specific factors can explain the remaining variance not accounted for by overall L2 grit. As the bifactor CFA was the more parsimonious model and exhibited slightly more fit than the bifactor ESEM, bifactor CFA was finally selected as the optimal model to represent the factor structure of L2 grit. Together, we identified the bifactor CFA as the best model among four alternative models to represent the factor structure of L2 grit. This finding was in line with Alamer (2021) who found bifactor CFA as the more optimal model than CFA among Saudi university students. The significance of the findings of the current study lies in the fact that we assess the factor structure in a more comprehensive manner, comparing four measurement models among Chinese high school students.

To the best of our knowledge, the current study is the first to test measurement invariance of L2 grit across gender, which was conducted drawing on the selected bifactor CFA model. The L2 grit scale was invariant across gender even in the most restrictive invariance model – strict invariance when modeled with bifactor approach. This means that the L2 grit scale assessed by bifactor CFA model is equivalent across gender with regards to factor structure, factor loadings, and item intercepts. This finding confirmed that the L2 grit scale based on the bifactor CFA model has identical theoretical structure across gender, and it can be used to yield meaningful results when comparing mean differences or other structural parameters across gender. Assessment of invariance is important as it provide statistical evidence of items functioning equivalently across groups and make inter-group comparison valid (Kline, 2013).

The predictive validity of L2 grit was tested by examining the relations of L2 grit with language achievements based on the bifactor CFA model. We found that general L2 grit, and its two specific dimensions (i.e., perseverance of effort and consistency of interest) were all predictive of language achievement at T1 (i.e., 2 weeks later). In other words, students with higher overall grit, consistency of interest, or perseverance of effort are more likely to score higher in language test. This finding supports the previous result in various L2 domains (e.g., Sudina et al., 2020; Teimouri et al., 2020; Khajavy, 2021; Liu and Wang, 2021). In addition, we found that the two dimensions had predictive power over and above the general L2 grit, which was in line with Alamer (2021) that examined the role of grit in vocabulary learning. This means that the two factors can predict language achievement after controlling for the effect of overall L2 grit. This further supports the bifactor CFA model adopted in the current study.

Interestingly, consistency of interest showed stronger predictive power than perseverance of effort in L2 achievement. This contrasts with the findings that reported a higher predictive power of perseverance of effort than consistency of interest in meta-analyses studies with domain-general grit (Credé et al., 2017; Lam and Zhou, 2019, 2022) and with the finding in domain-specific grit (Teimouri et al., 2020). However, the finding supports the results of Thorsen et al. (2021) with domain-general grit and Sudina and Plonsky (2021) with domain-specific grit. This suggests that the relative importance of the two dimensions in L2 achievement may be moderated by other learner internal or contextual variables. For example, the genuine interest and desire to acculturate in the targe culture in second language context can be a more powerful driving force than the instrumental need to pass an exam or further education in foreign language classroom setting. Additionally, the impacts of consistency of interest and perseverance of effort on language achievement can be moderated by motivational factors such as autonomous or controlled motivational orientations, as revealed in Alamer (2022b).

The present study is unique in that the enduring effect of grit including global grit, consistency of interest and perseverance of effort on language achievement was tested. We found that the general factor of grit and one factor of consistency of interest demonstrated small but significant predictive power in language achievement at T2 (after 6 months) after controlling for their effect on achievement at T1. This trivial long-term effect indicates that initial levels of global grit and consistency of interest affect the language achievement at T2 primarily through language achievement at T1. In other words, those who endorsed higher levels of global grit and consistency of interest tend to score higher at T1 which in turn have a beneficial effect on language achievement at T2. This small direct effect of global grit and consistency of interest on language achievement at T2 may also indicate that students’ initial levels of grit including consistency of interest and perseverance of effort may attenuate and therefore their effect on language achievement. As indicated by Alamer (2022b), students can endorse an initial high level of single language interest, but less so later on. However, the findings support the conceptualization of grit emphasizing the long-term component in the initial grit conceptualization (Duckworth et al., 2007; Duckworth and Quinn, 2009). As language learning is a long-term endeavor and needs persistent effort from the students to succeed, it is not surprising that long-term passion and perseverance can have a lasting positive effect on language achievement. The finding is also in line with the study by Alamer (2022b) that specifically focused on single language interest and found its predictive effect on language achievement after one academic year. Overall, the small but significant long-term effect of grit on language achievement was confirmed in the present study, supporting the grit hypothesis (Duckworth et al., 2007; Duckworth and Quinn, 2009). The small effect may indicate that other factors may be moderating its role, thus future research may need to incorporate other moderating factors to give a clearer picture of how grit interacts with other factors and jointly affect language achievement.

## Conclusion

The current study examined the validity of the L2 grit scale in Chinese senior high school EFL context. Among the four models tested (i.e., CFA, bifactor CFA, ESEM, bifactor ESEM). CFA and ESEM did not reach acceptable fit to the data whereas bifactor CFA and bifactor ESEM showed excellent fit with all four models showing acceptable composite reliabilities. More specifically, ESEM with partial fit demonstrated better fit than CFA with complete non-fit and bifactor CFA showed slightly better fit than bifactor ESEM. Thus, due to its more fit and parsimony, bifactor CFA was selected for further analysis. Then, measurement invariance of the bifactor CFA model across gender was tested. The findings supported the measurement equivalence across gender. That is, the model can be used to compare statistical results such as means and regression coefficient across gender. Furthermore, we tested the predictive validity of the bifactor ESEM model and revealed that all factors including one general factor (grit) and specific factors (perseverance of effort and consistency of interest) can predict language achievement at T1 (2 weeks later). Finally, we found the lasting positive impact of the L2 grit and consistency of effort on the language achievement at T2 (6 months later) even after controlling for the effect of T1.

## Implications and limitations

Based on the findings of the current study, we provide both methodological and pedagogical implications. For methodological implication, we suggest that future research, when faced with poor fit employing CFA to test a model structure, can use other more advanced alternative models including ESEM, bifactor CFA, and Bifactor ESEM. In SLA field, it is a common practice to use CFA model to test the construct validity, which is difficult to converge as a result of its over-restricted zero-correlation from items to non-target factors. In this situation, researchers tend to modify the model by correlating multiple measurement errors or deleting items. This was again viewed as pure data-driven and untheoretical. The current study provides more recent and advanced alternatives (i.e., bifactor CFA, ESEM, and bifactor ESEM) to compare the model fits for the ultimate purpose of identifying the optimal model within a theoretical framework. As found in the current study, all three alternative models showed better fit than traditional CFA model.

ESEM that allows cross-loadings showed better fit and smaller correlation between factors than CFA, indicating that allowing cross-loadings in a model can not only increase model fit but also reduce the inflated correlation between the factors. Two bifactor models (i.e., bifactor CFA and bifactor ESEM) demonstrated excellent fit to the data. This indicates the importance of incorporating both general factor and specific factors in L2 grit. This was evidenced by the findings of the current study that two factors produced predictive power over and above general grit. This is important as ignoring the existence of the general factor in addition to the specific factors may result in biased estimations such as overestimation of cross-loadings or correlations between factors. In addition, measurement invariance of L2 grit across gender was confirmed based on bifactor CFA, indicating that future research could use this model of L2 grit to compare structural parameters or means differences across gender.

For pedagogical implication, language teachers and educators are recommended to employ interventions to enhance students’ grit levels. As found in the current study, L2 grit and especially its component of consistency of interest can be influential not only in the short-term but also long-term language learning achievements. For example, language teachers are suggested to make students aware of the long arduous process of language learning, during which they are necessarily faced with obstacles and setbacks. Students need to set a long-term goal and keep sustained interest and work continuously towards these goals even in the face of difficulties and discouragements to finally achieve their ideal language proficiency. In addition, teacher should tell students that the talented students may not necessarily succeed in language learning, but instead those who showed interest and effort can be a more robust predictor of language success. Teachers may need to encourage growth mindset of students; when students believe that language ability improves with persistent effort and interest, they are more likely to show grit in the face of difficulties and adversities in language learning. Finally, giving examples of how celebrities succeeded in language learning through continuous passion and effort may help improve students’ grit level and subsequent language achievement (Liu et al., 2021).

There are some limitations. First, the current study adopted a homogenous group as our research sample, who have a similar educational background, similar age from the same geographical location. Thus, this may affect the generalizability of the research findings. Future research is suggested to use a more heterogeneous group to test whether these results hold for other educational, cultural, and geographical backgrounds with a wide range of age. In addition, other models can be tested such as second-order ESEM and set-ESEM to provide more options for the model test. Third, other related variables such as Big Five conscientiousness can be included in future studies to provide discriminant validity of the construct. Fifth, the L2 grit scale used in the present study has some methodological limitations, which have been discussed and addressed by prior studies (e.g., Oxford and Khajavy, 2021; Shirvan and Alamer, 2022).

## Data availability statement

The raw data supporting the conclusions of this article will be made available by the authors, without undue reservation.

## Ethics statement

The studies involving human participants were reviewed and approved by School of Foreign Languages, Shandong University, China. Written informed consent to participate in this study was provided by the participants’ legal guardian/next of kin.

## Author contributions

EL and SB collected and analyzed the data and wrote the first draft of the manuscript. EL and JW reviewed and revised the manuscript. All authors contributed to the article and approved the submitted version.

## Funding

This work was supported as a Major Project of the Chinese National Social Science Foundation (Grant No. 17AYY022).

## Conflict of interest

The authors declare that the research was conducted in the absence of any commercial or financial relationships that could be construed as a potential conflict of interest.

## Publisher’s note

All claims expressed in this article are solely those of the authors and do not necessarily represent those of their affiliated organizations, or those of the publisher, the editors and the reviewers. Any product that may be evaluated in this article, or claim that may be made by its manufacturer, is not guaranteed or endorsed by the publisher.

## Footnotes

^1^Robins, S. (2019). Academic achievement and retention among ESL learners: A study of grit in an online context (Unpublished doctoral dissertation). University of West Georgia.
