# Supplementary material for: Validation of L2 grit among Chinese EFL high school students and its enduring effect on achievements: A bifactor model approach
Source: Front Psychol. 2022 Sep 29;13:971495. doi: 10.3389/fpsyg.2022.971495 (PMC9559737; doi:10.3389/fpsyg.2022.971495)
Supplement: Supplementary file 1 [file Data_Sheet_1.docx]

# Supplementary material

# Appendix 1- Research materials

To what extent do you agree with the following statements?

Strongly Disagree/Disagree/Undecided/Agree/Strongly Agree.

| 1. 我是一个勤奋的英语学习者。 | I am a diligent English language learner. |
| --- | --- |
| 1. 对英语而言，我是一个努力学习的人。 | When it comes to English, I am a hard-working learner. |
| 1. 只要我决定学好英语，任何事情都不能阻止我。 | Now that I have decided to learn English, nothing can prevent me from reaching this goal. |
| 1. 任何事情都不能阻止我英语的进步。 | I will not allow anything to stop me from my progress in learning English. |
| 1. 我会花费大量的时间和精力来提高我的英语薄弱点。 | I put much time and effort into improving my English language weaknesses. |
| 1. 我对英语的兴趣一直都有变化。 | My interests in learning English change from year to year. |
| 1. 我对英语已经失去兴趣了。 | I think I have lost my interest in learning English. |
| 1. 我没有像以前那样对英语感兴趣了。 | I am not as interested in learning English as I used to be. |
| 1. 我曾经对英语感兴趣，但最近对英语失去兴趣了。 | I was obsessed with learning English in the past but have lost interest recently. |

# Appendix 2 - Mplus code

1. Confirmatory factor analysis (CFA)

Usevariables are n1 n2 n3 n4 n5 n6 n7 n8 n9;

MISSING ARE ALL (-999);

Analysis:

ESTIMATOR IS MLR;

Model:

PE by n1 n2 n3 n4 n5;

CI by n6 n7 n8 n9;

Output:

STDYX modindices;

1. Bifactor confirmatory factor analsis (Bifactor CFA)

Usevariables are n1 n2 n3 n4 n5 n6 n7 n8 n9;

MISSING ARE ALL (-999);

Analysis:

ESTIMATOR IS MLR;

Model:

PE by n1* n2 n3 n4 n5;

CI by n6* n7 n8 n9;

Grit by n1* n2 n3 n4 n5 n7 n8 n9;

PE with CI@0;

Grit with CI@0;

Grit with PE@0;

Grit@1; PE@1; CI@1;

Output:

STDYX;

1. Exploraotry structural equation model (ESEM)

Usevariables are n1 n2 n3 n4 n5 n6 n7 n8 n9;

MISSING ARE ALL (-999);

ANALYSIS:

ESTIMATOR=MLR;

ROTATION=TARGET;

MODEL:

PE by n1-n5

n6-n9~0 (*1);

CI by n6-n9

n1-n5~0 (*1);

OUTPUT:

stdyx;

tech4;

mod(10);

1. Bifactor Exploratory factor analsyis (Bifactor ESEM)

Usevariables are n1 n2 n3 n4 n5 n6 n7 n8 n9;

MISSING ARE ALL (-999);

ANALYSIS:

ESTIMATOR=MLR;

ROTATION=TARGET (orthogonal);

MODEL:

PE by n1-n5

n6-n9~0 (*1);

CI by n6-n9

n1-n5~0 (*1);

OUTPUT:

stdyx;

tech4;

1. Structural bifactor CFA model predciting subsequent three langauge achievements

Usevariables are n1 n2 n3 n4 n5 n6 n7 n8 n9 Score1 Score2 Score3;

MISSING ARE ALL (-999);

Analysis:

ESTIMATOR IS MLR;

Model:

PE by n1* n2 n3 n4 n5;

CI by n6* n7 n8 n9;

Grit by n1* n2 n3 n4 n5 n6 n7 n8 n9;

PE with CI@0;

Grit with CI@0;

Grit with PE@0;

Grit@1; PE@1; CI@1;

Score1 on Grit PE CI;

Score2 on Grit PE CI Score1;

Score3 on Grit PE CI Score1 Score2;

Output:

STDYX;
